# Impact of chest pain center quality control indicators on mortality risk in ST-segment elevation myocardial infarction patients: a study based on Killip classification

**DOI:** 10.3389/fcvm.2023.1243436

**Published:** 2024-01-03

**Authors:** Lingling Zhang, Jianping Zeng, Haobo Huang, Yunlong Zhu, Ke Peng, Cai Liu, Fei Luo, Wenbin Yang, Mingxin Wu

**Affiliations:** ^1^ Department of Cardiology, Xiangtan Central Hospital, Xiangtan, China; ^2^ Chest Pain Centre, Xiangtan Central Hospital, Xiangtan, China; ^3^ Department of Scientific Research, Xiangtan Central Hospital, Xiangtan, China; ^4^ Medical Department, Xiangtan Central Hospital, Xiangtan, China; ^5^ Graduate Collaborative Training Base of Xiangtan Central Hospital, Hengyang Medical School, University of South China, Hengyang, Hunan, China; ^6^ Department of Cardiology, the Second Xiangya Hospital of Central South University, Changsha, Hunan, China

**Keywords:** chest pain center, CPC, quality control, STEMI, primary percutaneous coronary intervention, PPCI, door-to-balloon, D-to-B

## Abstract

**Background:**

Despite the crucial role of Chest pain centers (CPCs) in acute myocardial infarction (AMI) management, China's mortality rate for ST-segment elevation myocardial infarction (STEMI) has remained stagnant. This study evaluates the influence of CPC quality control indicators on mortality risk in STEMI patients receiving primary percutaneous coronary intervention (PPCI) during the COVID-19 pandemic.

**Methods:**

A cohort of 664 consecutive STEMI patients undergoing PPCI from 2020 to 2022 was analyzed using Cox proportional hazards regression models. The cohort was stratified by Killip classification at admission (Class 1: *n* = 402, Class ≥2: *n* = 262).

**Results:**

At a median follow-up of 17 months, 35 deaths were recorded. In Class ≥2, longer door-to-balloon (D-to-B) time, PCI informed consent time, catheterization laboratory activation time, and diagnosis-to-loading dose dual antiplatelet therapy (DAPT) time were associated with increased mortality risk. In Class 1, consultation time (notice to arrival) under 10 min reduced death risk. In Class ≥2, PCI informed consent time under 20 min decreased mortality risk.

**Conclusion:**

CPC quality control metrics affect STEMI mortality based on Killip class. Key factors include time indicators and standardization of CPC management. The study provides guidance for quality care during COVID-19.

## Introduction

### Background on cardiovascular disease

Cardiovascular disease (CVD) remains a primary cause of global mortality (1), accounting for over 40% of deaths in China (2). Among various forms of CVD, acute myocardial infarction (AMI) represents a prevalent and severe condition with a notably high mortality rate (3), posing a significant global public health concern. In developed countries, epidemiological studies have shown a decline in AMI incidence, hospitalization, and mortality rates (4–6). Conversely, in China, these rates are on the rise (7, 8), emphasizing the need to establish Chest Pain Centers (CPC) for optimizing AMI treatment quality and mortality reduction (9, 10).

### Impact of COVID-19 on STEMI treatment

The 2020 outbreak of the novel coronavirus led to a substantial decline in hospital admissions for ST-elevation myocardial infarction (STEMI) and associated treatment delays (11). This pandemic introduced unprecedented challenges for public health and healthcare systems (12). Medical professionals are now tasked with finding a balance between timely STEMI treatments and infection control measures, to curb the nosocomial transmission of COVID-19 among healthcare workers and other susceptible individuals (11).

### China's response: the role of CCPC

The Chinese Chest Pain Center (CCPC) certification is the third professional association certification, following similar systems in the United States and Germany (13). The quality control indicators set by the CPC serve as a national benchmark for quality assurance and continuous improvement across China. To counteract the adverse effects of the COVID-19 pandemic on STEMI healthcare, a nationwide Quality Improvement (QI) initiative has been implemented by the National CPC (14).

### Focus of the current study

While reducing D-to-B time significantly enhances outcomes for STEMI patients (15, 16), it's important to note that many factors, not just D-to-B time, influence STEMI patient prognosis. During the COVID-19 pandemic, hesitancy in seeking medical care, healthcare resource constraints, and delays due to comprehensive testing have led to prolonged D-to-B times. This study aims to provide a holistic view of STEMI care. Beyond the critical indicators highlighted by CPC guidelines, we analyze the entire diagnostic and treatment process—from the onset of STEMI to patient discharge. Our findings aspire to create a strong foundation for refining CPC establishments and implementing multifaceted interventions, ultimately improving outcomes for STEMI patients.

## Methods

### Ethics and informed consent

This research protocol was approved by the Ethics Committee of Xiangtan Central Hospital (Xiangtan, China; Ethics Approval No: 2023-02-001) and adhered to the principles outlined in the Helsinki Declaration. The study design was retrospective, involving the collection of clinical data without intervention in patient treatment plans, thus obviating the need for informed consent.

### Study design and population

**Figure 1:**
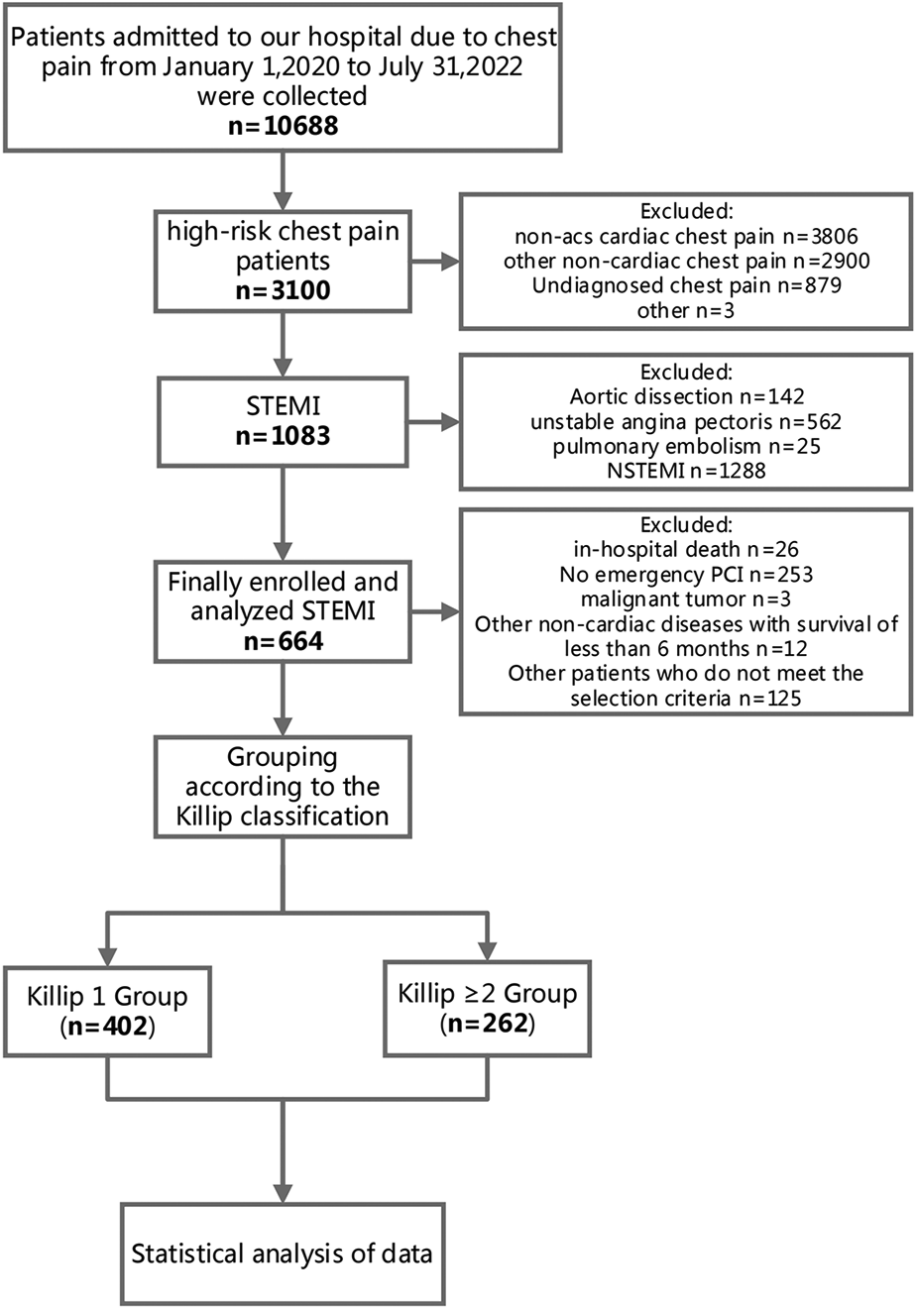
Flowchart of the study design and participant selection.

This retrospective observational study was conducted at a single center. We consecutively collected data from 10,688 patients with acute chest pain who sought treatment at our CPC between January 1, 2020, and July 31, 2022. Among them, 3,100 were identified as high-risk chest pain patients. Following the diagnostic criteria outlined in the “2019 Guidelines for the Diagnosis and Treatment of Acute ST-segment Elevation Myocardial Infarction,” we identified 1,083 patients with STEMI. According to the ACC/AHA STEMI management guidelines, which recommend treatment within 24 h of symptom onset, we finally included 664 STEMI patients who underwent emergency percutaneous coronary intervention (PCI) (Figure 1).

The inclusion criteria were as follows: (1) First-time occurrence of STEMI as defined by the guidelines (17); (2) Underwent emergency PCI; (3) Complete documentation of chest pain onset time. The exclusion criteria were: (1) Age under 18 years; (2) Missing relevant important data; (3) In-hospital mortality; (4) STEMI patients who did not undergo PCI; (5) Malignant tumors or non-cardiac diseases with an expected survival time of less than 6 months. Killip classification was utilized to assess the severity of STEMI, categorizing patients into two groups based on their Killip classification at admission: Killip class 1 and Killip class ≥2.

### Study methods

The data for this study were obtained from the hospital's medical record system and the CPC database, which encompassed demographic information of the study participants. Follow-up for all study participants extended until January 31, 2023, with a median clinical follow-up time of 17 months. A team comprising five experienced cardiovascular physicians and two nurses conducted on-site and telephone follow-ups and reviewed hospital visit records to ascertain patient outcome events. The primary endpoint for follow-up was all-cause mortality.

### Establishment and data collection of the CPC database

The CPC data were recorded in real-time by medical personnel from various departments involved in patient care, including network hospitals, pre-hospital emergency services, emergency departments, cardiology departments, and catheterization laboratories. The data were subsequently entered and reported on the CPC data platform using a chest pain timeline table. The accuracy and completeness of the data were ensured through review and auditing by a quality control team (comprising two nurses and two cardiologists) from the CPC.

### Primary outcome measures and definitions

#### Quality control indicators for the CPC

In addition to the quality control indicators specified by the CCPC, we incorporated supplementary indicators to comprehensively evaluate and analyze the study patients. These indicators encompassed various time intervals, including:first medical contact to first electrocardiogram (FMC-to-ECG), first medical contact to loading dose dual antiplatelet therapy (FMC-to-loading dose DAPT), Troponin report time, consultation time (notice to arrival), diagnosis to loading dose DAPT, diagnosis-to-the first intravenous heparin, door-to-balloon time (D-to-B), total ischemic time (onset-to-reperfusion), symptom onset to first medical contact time (S0-to-FMC), FMC inhospital-to-start reperfusion time, Diagnosis and treatment time in emergency department, FMC inhospital-to-notice consultation, FMC inhospital-to-notice consultation, Leave ED to arrive catheter lab (CL), PCI informed consent time.

### CL activation time

#### Quality control assessment program for the CPC

To account for the unique circumstances of the hospital, we developed a comprehensive quality control assessment program for the CPC. In addition to the individual case-based quality control indicators, we formulated specific assessment indicators for the main departments involved in the treatment of acute chest pain patients at the CPC. This allowed for a more precise evaluation of departmental involvement and compliance with the standards set by the CPC, enabling an assessment of their impact on patient prognosis.

### Statistical methods

We employed Cox proportional hazards regression models to examine the association between various quality control indicators of the CPC and outcomes such as all-cause mortality in STEMI patients undergoing emergency PCI. Normally distributed data were presented as mean ± standard deviation, skewed distributed data as median (interquartile range), and categorical variables as frequencies (percentages). Clinical characteristics between groups were compared using Student's *t*-test for continuous variables and chi-square test for categorical variables. Kaplan–Meier analysis was used to estimate cumulative event rates, and curve fitting was performed to identify inflection points. *P*-values were obtained using the Kruskal–Wallis rank-sum test for continuous variables and Fisher's exact probability test for count variables. Results were considered significant when *P* < 0.05. Statistical analysis was conducted using R software (version 4.2.0) and EasyStat software.

## Results

### Baseline characteristics comparison

Age and Underlying Diseases: Patients in the Killip ≥2 group were significantly older (65.04 ± 11.36 years vs. 60.56 ± 12.23 years, *P* < 0.001) and had a higher prevalence of conditions like atrial fibrillation, diabetes, stroke, and renal insufficiency than the Killip 1 group (*P* < 0.05).

Clinical Features: The Killip ≥2 group exhibited more in-hospital new-onset heart failure and a higher heart rate. They also had lower systolic blood pressure and showed inferior results for NT-proBNP, TnT, and LVEF.

Treatment Differences: The Killip ≥2 group saw more usage of treatments like spironolactone, anticoagulants, vasoactive drugs, positive inotropic drugs, IABP, and temporary pacemakers.

Quality Control Indicators: The Killip ≥2 group experienced longer times in various stages, such as FMC-to-loading dose DAPT time, diagnosis-to-loading dose DAPT time, and D-to-B time (Table 1).

**Table 1:** Baseline characteristics.

	Killip class 1 group (*n* = 402>)	Killip class ≥ 2 group (*n* = 262>)	Standardize diff.	*P*-value
Demographics				
Age, years	60.56 ± 12.23	65.04 ± 11.36	0.38 (0.22, 0.54)	<0.001
Female, *N* (%)	82 (20.40%)	67 (25.57%)	0.12 (−0.03, 0.28)	0.118
Obesity, *N* (%)	121 (30.10%)	63 (24.05%)	0.14 (−0.02, 0.29)	0.088
Hospitalization days	8.38 ± 3.13	9.86 ± 5.55	0.33 (0.17, 0.49)	<0.001
CCU hospitalization days	3.18 ± 1.63	3.74 ± 2.37	0.28 (0.12, 0.43)	<0.001
Total hospitalization expenses	38,130.44 ± 15,022.87	46,479.68 ± 25,313.23	0.40 (0.24, 0.56)	<0.001
Medical history, *N* (%)				
Current smoker	243 (60.45%)	135 (51.53%)	0.18 (0.02, 0.34)	0.023
Current drinker	58 (14.43%)	37 (14.12%)	0.01 (−0.15, 0.16)	0.912
Coronary heart disease	402 (100.00%)	261 (99.62%)	0.09 (−0.07, 0.24)	0.215
Hyperlipidemia	162 (40.30%)	91 (34.73%)	0.12 (−0.04, 0.27)	0.149
Hypertension	238 (59.20%)	144 (54.96%)	0.09 (−0.07, 0.24)	0.28
Atrial fibrillation	14 (3.48%)	38 (14.50%)	0.39 (0.24, 0.55)	<0.001
Diabetes mellitus	99 (24.63%)	87 (33.21%)	0.19 (0.03, 0.35)	0.016
Stroke	39 (9.70%)	47 (17.94%)	0.24 (0.08, 0.40)	0.002
Renal insufficiency	33 (8.21%)	65 (24.81%)	0.46 (0.30, 0.62)	<0.001
Clinical conditions at admission				
New heart failure in hospital	3 (0.75%)	17 (6.49%)	0.31 (0.15, 0.47)	<0.001
Systolic blood pressure, mmHg	131.02 ± 21.10	121.25 ± 28.28	0.39 (0.23, 0.55)	<0.001
Heart rate, bpm	78.68 ± 13.72	83.66 ± 20.26	0.29 (0.13, 0.44)	<0.001
NT-proBNP, pg/ml	1,003.05 ± 2,745.66	2,845.30 ± 4,328.99	0.51 (0.35, 0.67)	<0.001
TnT, ng/ml	4.48 ± 3.40	5.73 ± 3.71	0.35 (0.20, 0.51)	<0.001
Cr, µmol/L	82.84 ± 81.27	90.44 ± 38.29	0.12 (−0.04, 0.28)	0.158
LVEF, %	53.19 ± 7.33	47.21 ± 9.71	0.69 (0.53, 0.85)	<0.001
Treatment, *N* (%)				
CCB	53 (13.18%)	24 (9.16%)	0.13 (−0.03, 0.28)	0.113
Beta-blocker	362 (90.05%)	228 (87.02%)	0.10 (−0.06, 0.25)	0.226
ACEI	139 (34.58%)	86 (32.82%)	0.04 (−0.12, 0.19)	0.641
ARB	143 (35.57%)	80 (30.53%)	0.11 (−0.05, 0.26)	0.179
ARNI	154 (38.31%)	115 (43.89%)	0.11 (−0.04, 0.27)	0.152
SGLT2i	26 (6.47%)	28 (10.69%)	0.15 (−0.00, 0.31)	0.052
Spironolactone	79 (19.65%)	73 (27.86%)	0.19 (0.04, 0.35)	0.014
Statins	400 (99.50%)	258 (98.47%)	0.10 (−0.05, 0.26)	0.171
Antiplatelet Drugs	400 (99.50%)	259 (98.85%)	0.07 (−0.08, 0.23)	0.345
Anticoagulant drugs	62 (15.42%)	73 (27.86%)	0.31 (0.15, 0.46)	<0.001
Vasoactive drugs	15 (3.73%)	77 (29.39%)	0.74 (0.57, 0.90)	<0.001
Positive inotropic drugs	4 (1.00%)	32 (12.21%)	0.46 (0.31, 0.62)	<0.001
Vasodilators	340 (84.58%)	201 (76.72%)	0.20 (0.04, 0.36)	0.011
IABP	6 (1.49%)	27 (10.31%)	0.38 (0.22, 0.54)	<0.001
Temporary cardiac pacemaker	20 (4.98%)	26 (9.92%)	0.19 (0.03, 0.35)	0.014
ECMO	2 (0.50%)	1 (0.38%)	0.02 (−0.14, 0.17)	0.828
Left ventricular assist device	2 (0.50%)	1 (0.38%)	0.02 (−0.14, 0.17)	0.828
IVUS	19 (4.7%)	8 (3.1%)	0.1 (−0.1, 0.2)	0.286
CPC quality control index, min				
FMC-to-ECG	4.26 ± 3.50	4.49 ± 3.53	0.07 (−0.09, 0.22)	0.397
FMC-to-loading dose DAPT	31.58 ± 29.38	39.73 ± 35.08	0.25 (0.10, 0.41)	0.001
Diagnosis-to-loading dose DAPT	8.82 ± 8.31	10.44 ± 9.78	0.18 (0.02, 0.33)	0.023
Diagnosis-to-the first intravenous heparin	10.92 ± 4.48	11.59 ± 4.06	0.16 (−0.00, 0.31)	0.053
Troponin report time	17.59 ± 2.49	17.55 ± 2.25	0.02 (−0.14, 0.17)	0.829
Consultation time (notice to arrival)	3.36 ± 1.82	3.30 ± 1.69	0.03 (−0.12, 0.19)	0.673
D-to-B	67.87 ± 25.17	72.15 ± 29.21	0.16 (0.00, 0.31)	0.045
Total ischemic time (onset-to-reperfusion)	383.69 ± 395.27	475.23 ± 665.85	0.17 (0.01, 0.32)	0.027
SO-to-FMC	257.83 ± 368.26	318.34 ± 633.57	0.12 (−0.04, 0.27)	0.121
FMC inhospital-to-start reperfusion	22.47 ± 17.82	25.50 ± 20.74	0.16 (0.00, 0.31)	0.045
Diagnosis and treatment time in ED	30.41 ± 23.34	28.89 ± 16.93	0.07 (−0.08, 0.23)	0.364
FMC inhospital-to-notice consultation	11.50 ± 15.17	10.59 ± 11.34	0.07 (−0.09, 0.22)	0.407
Leave ED to arrive CL	17.30 ± 23.30	22.19 ± 27.98	0.19 (0.03, 0.35)	0.015
PCI informed consent time	11.07 ± 9.32	11.85 ± 12.35	0.07 (−0.08, 0.23)	0.353
CL activation time	13.49 ± 11.01	14.29 ± 9.91	0.08 (−0.08, 0.23)	0.343
Outcome event				
Death	8 (1.99%)	27 (10.31%)	0.35 (0.19, 0.51)	<0.001

NT-proBNP, N-terminal pro-B type natriureti peptide; TnT, Troponin T; Cr, creatinine; LVEF, left ventricular ejection fraction; CCB, calcium antagonist; ACEI, angiotensin-converting enzyme inhibitors; ARB, angiotensin receptor blockers; ARNI, angiotensin receptor—enkephalase inhibitors; SGLT2i, sodium-dependent glucose transporters 2; IABP, intraaortic balloon counter ppulsation; ECMO, extracorporeal membrane oxygenation; IVUS, intravenous ultrasound; CPC, Chest Pain Center; FMC, first medical contact; ECG, electrocardiogram; DAPT, dual antiplatelet therapy; ACS, acute coronary syndrome; STEMI, St-segment elevation myocardial infarction; D-to-B,> door-to-balloon; SO, symptom onset; ED, emergency department; CL, catheter lab; PCI, percutaneous transluminal coronary intervention; CCU, coronary care unit.

### Clinical outcomes based on quality control indicators in the CPC

**Figure 2:**
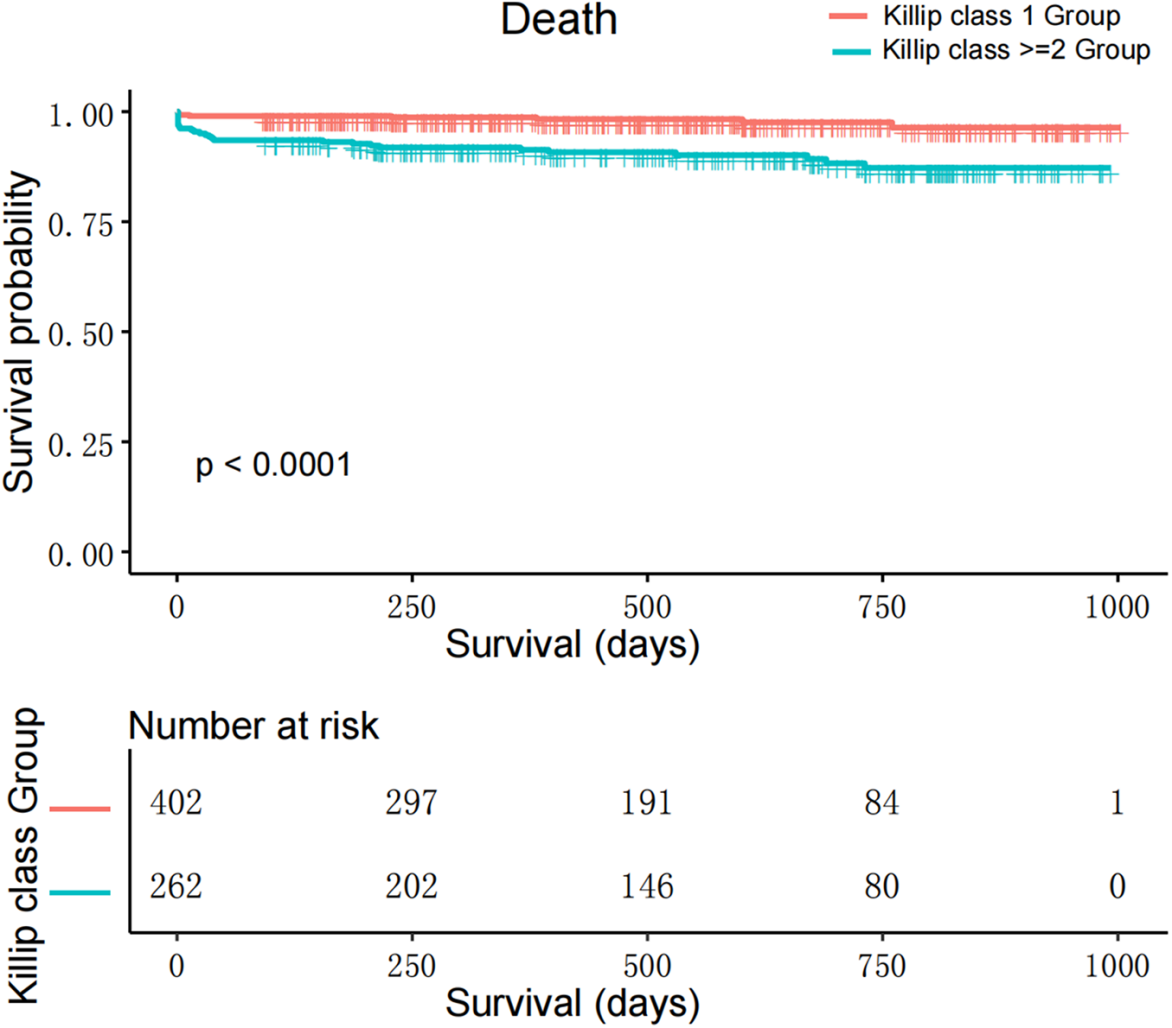
Kaplan–Meier survival curves for patients with STEMI undergoing emergency PCI, categorized by Killip class.

Mortality Risk in Killip Groups: After adjusting, the Killip ≥2 group faced a higher risk of mortality than the Killip 1 group (HR: 2.56; 95% CI: 1.06–6.15; *P* < 0.05) (Table 2, Figure 2). See Supplementary Table 1 for detailed baseline characteristics and Table 3 for univariate and multivariable analysis.

**Table 2:** Results of a multivariate Cox proportional hazards model for the effect of admission Killip classification on death in STEMI patients undergoing emergency PCI.

	Non-adjusted Hazard ratio (95% CI)	Adjust I Hazard/Risk ratio (95% CI)	Adjust II Hazard/Risk ratio (95% CI)
Death			
Killip class 1 Group	Ref.	Ref.	Ref.
Killip class ≥2 Group	4.93 (2.24, 10.87)‡	4.15 (1.87, 9.20)‡	2.56 (1.06, 6.15)§

PCI,> percutaneous transluminal coronary intervention; STEMI,> St-segment elevation myocardial infarction; CI,> conﬁdence interval; CCU,> coronary care unit.

Non-adjusted model adjust for: None.

Adjust I model adjust for: sex (woman 0/man 1); age.

Adjust II model adjust for: sex; age; current smoker; hyperlipidemia; hypertension; atrial fibrillation/atrial flutter; diabetes mellitus; stroke; renal insufficiency; admission systolic blood pressure; admission heart rate; maximum NT-proBNP; maximum troponin.

^‡^*P* < 0.001. ^§^*P* < 0.05.

**Table 3:** Single factor analysis and multivariate Cox proportional risk analysis of quality control indicators and death after grouping by Killip classification.

	Non-adjusted Hazard ratio (95% CI)		Multivariable adjusted Hazard ratio (95% CI)	
	Killip class 1 Group	Killip class ≥2 Group	Killip class 1 Group	Killip class ≥2 Group
Quality control indexs as continuous variables				
FMC-to-ECG	1.059 (0.893, 1.256)	0.982 (0.878, 1.098)	1.022 (0.851, 1.227)	1.004 (0.899, 1.121)
FMC-to-loading dose DAPT	0.960 (0.908, 1.014)	1.004 (0.995, 1.014)	0.938 (0.874, 1.007)	1.004 (0.993, 1.015)
diagnosis-to-loading dose DAPT	0.889 (0.765, 1.033)	**1.043 (1.008, 1.079)§**	0.859 (0.718, 1.028)	**1.063 (1.021, 1.106)§**
Diagnosis-to-the first intravenous heparin	0.884 (0.732, 1.067)	1.055 (0.973, 1.144)	0.900 (0.742, 1.092)	1.049 (0.961, 1.146)
Troponin report time	0.970 (0.721, 1.304)	1.048 (0.912, 1.203)	0.953 (0.700, 1.297)	1.048 (0.883, 1.243)
Consultation time (notice to arrival)	1.275 (0.992, 1.640)	**1.263 (1.054, 1.514)§**	1.237 (0.954, 1.604)	1.203 (0.974, 1.485)
D-to-B time	1.002 (0.975, 1.029)	**1.018 (1.007, 1.030)§**	0.992 (0.961, 1.025)	**1.021 (1.006, 1.035)§**
Total ischemic time (onset-to-reperfusion)	1.001 (0.999, 1.002)	1.000 (0.999, 1.001)	1.001 (0.999, 1.002)	1.000 (0.999, 1.001)
SO-to-FMC	1.001 (0.999, 1.002)	1.000 (0.999, 1.001)	1.001 (0.999, 1.002)	1.000 (0.999, 1.001)
FMC inhospital-to-start reperfusion	1.019 (0.990, 1.050)	1.006 (0.989, 1.023)	1.018 (0.983, 1.055)	1.010 (0.991, 1.030)
Diagnosis and treatment time in ED	1.005 (0.983, 1.027)	1.003 (0.981, 1.026)	1.011 (0.985, 1.039)	1.004 (0.977, 1.032)
FMC inhospital-to-notice consultation	1.011 (0.985, 1.038)	0.971 (0.911, 1.035)	1.020 (0.990, 1.052)	0.991 (0.927, 1.060)
Leave ED to arrive CL	0.993 (0.954, 1.032)	1.006 (0.995, 1.017)	0.973 (0.927, 1.021)	1.011 (0.999, 1.024)
PCI informed consent time	0.952 (0.861, 1.053)	**1.033 (1.014, 1.051)‡**	0.914 (0.815, 1.026)	**1.036 (1.015, 1.059)§**
CL activation time	0.950 (0.868, 1.040)	**1.038 (1.006, 1.070)§**	0.945 (0.875, 1.021)	**1.045 (1.004, 1.088)§**
Whether quality control indicators meet the standard				
FMC-to-ECG < 10 min	0.534 (0.066, 4.343)	1.389 (0.329, 5.866)	0.702 (0.067, 7.309)	1.136 (0.256, 5.051)
FMC-to-loading dose DAPT < 30 min	1.989 (0.399, 9.910)	0.625 (0.292, 1.336)	2.525 (0.405, 15.723)	0.591 (0.257, 1.358)
Diagnosis-to-loading dose DAPT < 10 min	5.003 (0.608, 41.176)	0.539 (0.252, 1.152)	5.758 (0.603, 54.997)	0.497 (0.214, 1.156)
Diagnosis-to-the first intravenous heparin < 10 min	1.721 (0.321, 9.218)	1.180 (0.351, 3.967)	1.531 (0.250, 9.359)	0.755 (0.195, 2.922)
Troponin report time < 20 min	NA	0.252 (0.059, 1.065)	NA	0.408 (0.060, 2.783)
Consultation time (notice to arrival) <10 min	**0.080 (0.010, 0.650)§**	0.193 (0.026, 1.428)	**0.077 (0.006, 0.958)§**	0.204 (0.019, 2.202)
D-to-B < 90 min	1.505 (0.185, 12.233)	0.553 (0.248, 1.232)	1.930 (0.221, 16.873)	0.526 (0.210, 1.321)
Total ischemic time (onset-to-reperfusion) < 120 min	NA	1.151 (0.346, 3.829)	NA	1.066 (0.301, 3.778)
SO-to-FMC < 90 min	NA	0.741 (0.332, 1.652)	NA	0.622 (0.259, 1.496)
FMC inhospital-to-start reperfusion < 30 min	0.394 (0.094, 1.652)	0.750 (0.328, 1.715)	0.509 (0.104, 2.485)	0.706 (0.275, 1.815)
Diagnosis and treatment time in ED <30 min	0.528 (0.130, 2.155)	0.758 (0.354, 1.619)	0.409 (0.083, 2.013)	0.643 (0.277, 1.496)
FMC inhospital-to-notice consultation < 15 min	0.378 (0.090, 1.583)	0.749 (0.284, 1.980)	0.297 (0.060, 1.476)	0.563 (0.186, 1.704)
Leave ED to arrive CL < 20 min	0.965 (0.228, 4.087)	0.690 (0.324, 1.469)	1.991 (0.354, 11.184)	0.584 (0.249, 1.368)
PCI informed consent time < 20 min	1.738 (0.213, 14.156)	**0.324 (0.150, 0.697)§**	4.020 (0.380, 42.566)	**0.258 (0.099, 0.675)§**
CL activation time < 30 min	NA	1.653 (0.224, 12.194)	NA	1.443 (0.173, 12.058)

CI,> confidence interval; MACE,> major adverse cardiac events; CEP,> composite endpoint; CCU,> coronary care unit; FMC,> first medical contact; ECG,> electrocardiogram; DAPT,> dual antiplatelet therapy; D-to-B,> door-to-balloon; SO,> symptom onset; ED,> emergency department; CL,> catheter lab; PCI,> percutaneous transluminal coronary intervention; NA,> not available.

Bold represent significant values (*P* < 0.05).

^‡^*P* < 0.001. ^§^*P* < 0.05.

**Figure 3:**
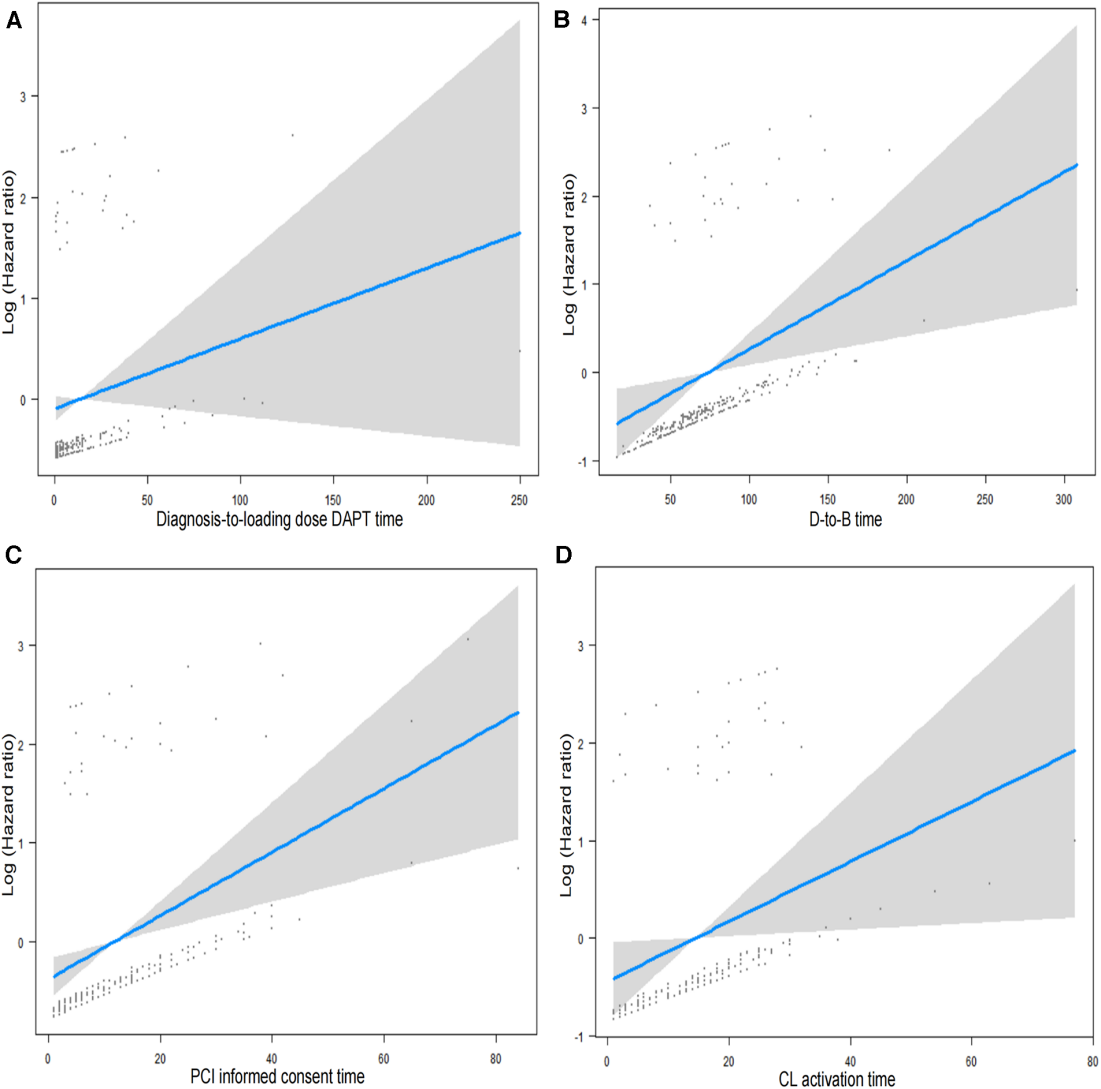
Relationship between time-related metrics and mortality in STEMI patients undergoing emergency PCI: (**A**) displays the relationship between the time from diagnosis to the administration of loading dose dual antiplatelet therapy (DAPT) and patient mortality. (**B**) Demonstrates the association between door-to-balloon (D-to-B) time, which represents the time from the patient's arrival at the hospital to the opening of the blocked artery, and patient mortality. (**C**) Shows the correlation between the time taken to obtain informed consent for PCI and patient mortality. (**D**) Presents the relationship between Cath Lab activation time, the duration from the decision of performing PCI to the actual procedure initiation, and patient mortality.

Impacts of Time Delays: In the Killip ≥2 group, delays like increased D-to-B time, informed consent time for PCI, catheterization laboratory activation time, and time from confirmed diagnosis-to-loading dose DAPT were associated with higher mortality risks (Table 3, Figure 3).

**Figure 4:**
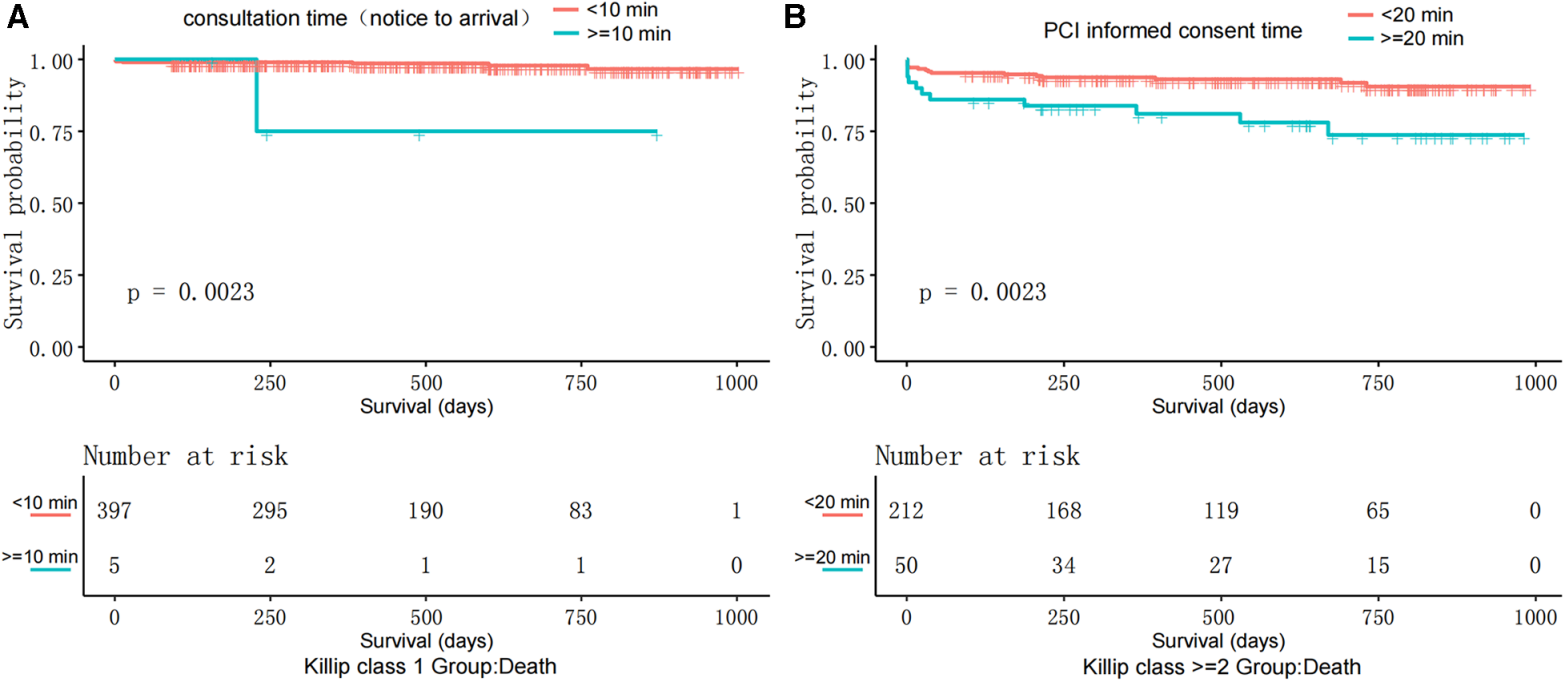
Kaplan–Meier survival curves: (**A**) displays the survival probability comparison between patients with Killip class I where the consultation time (notice to arrival) is less than 10 min and those with consultation time (notice to arrival) equal to or more than 10 min. (**B**) Illustrates the survival probability comparison among patients with Killip Class ≥2, where the time to obtain informed consent for PCI is less than 20 min and those with PCI informed consent time equal to or more than 20 min.

Beneficial Timeframes: In Killip 1, consultation time (notice to arrival) under 10 min reduced death risk by 92.3%. For Killip ≥2, an informed consent time (start-signature) under 20 min reduced death risk by 74.2% (Table 3, Figure 4).

### Clinical outcomes in different core departments

Protocols and Death Risk: Filling in the chest pain form as per the emergency department protocol reduced the death risk by 68.8%. Standardized writing of discharge records in the cardiology ward further decreased it by 92.9% (Supplementary Table 2).

## Discussion

### Objectives and main outcomes of the study

**Figure 5:**
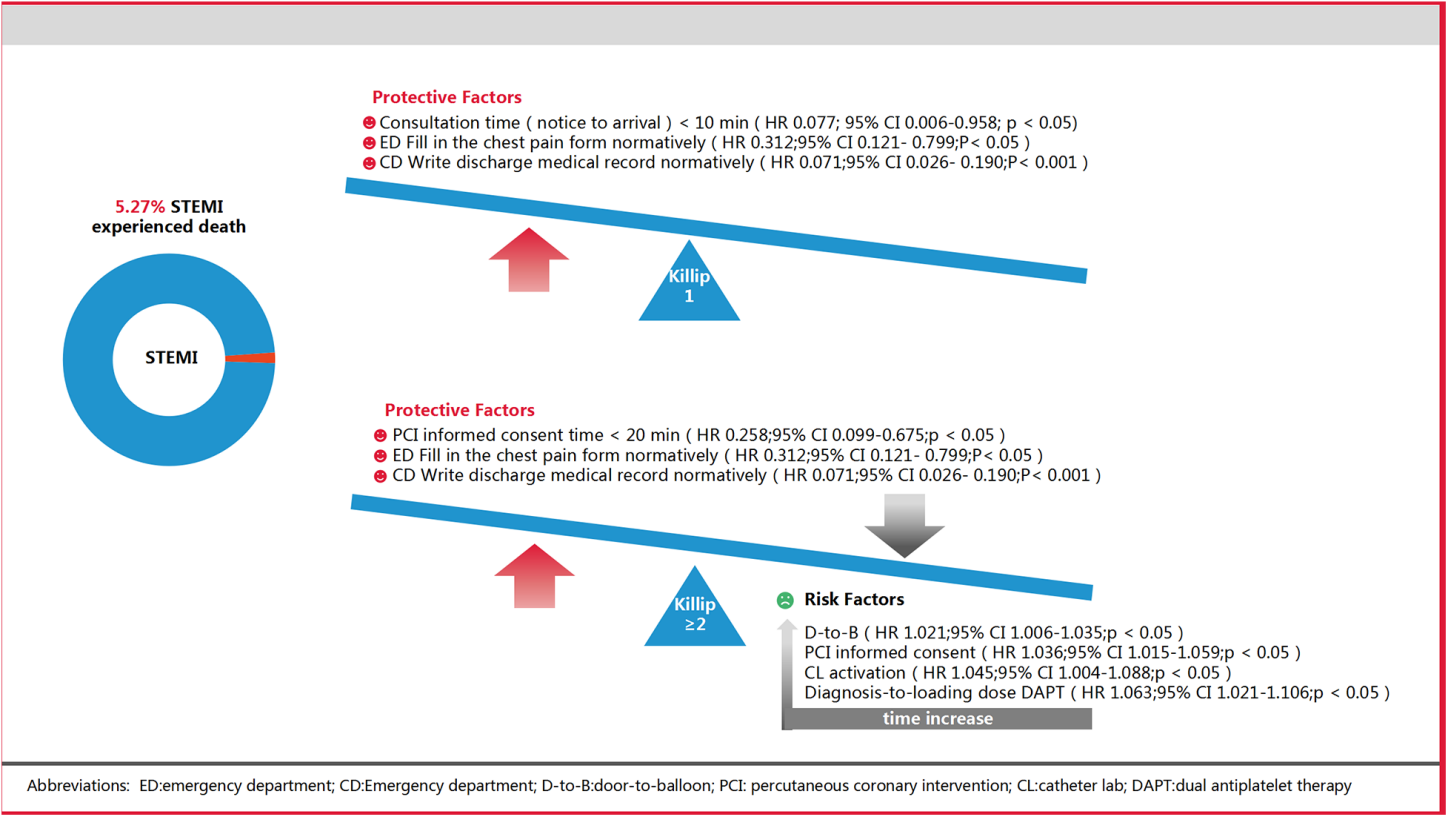
Core results.

This study was conducted to thoroughly evaluate the influence of multiple quality control indicators within the CPC on the mortality risk for STEMI patients who underwent PCI during the COVID-19 outbreak. The findings indicate that prolonged D-to-B time, PCI informed consent time, catheterization laboratory activation time, and the span from diagnosis to DAPT are strongly related to increased mortality risks for patients with Killip ≥2, but not as significantly for those classified under Killip 1 (Figure 5).

### Significance of time-sensitive interventions

The outcomes stress the value of prompt interventions and a fluid workflow within the CPC to better serve STEMI patients. Key aspects like the extended D-to-B time, informed PCI consent duration, catheterization lab activation period, and the time from confirmed ACS diagnosis to DAPT initiation, particularly in Killip ≥2 patients, show the critical importance of timely and coordinated efforts in handling high-risk patients.

### Comparative analysis with prior research

Compared to preceding research, our study aligns in asserting that establishing chest pain centers elevates AMI quality control and enhances its prognosis (18). The observed correlation between D-to-B time and patient mortality has been similarly emphasized in previous studies, which advocate for the urgency in the establishment of chest pain centers to upgrade the emergency cardiovascular disease management, especially regarding AMI (19). Like other countries, the routine STEMI protocol as recommended by Chest Pain Centers considers PPCI as the standard treatment (20). Our study illuminates that while prolonged D-to-B times escalate mortality risks for those under the Killip ≥2 classification, they don't significantly affect the Killip 1 group. This is in line with another retrospective study (21) and a cross-sectional study from 2018 to 2021 (22), both underscoring the importance of rapid PCI intervention.

### In-depth insights beyond previous studies

Our research distinguishes itself by offering new insights into key facets and quality control metrics within chest pain centers that can effectively reduce mortality rates. Unlike prior studies that broadly hinted at the potential of chest pain centers to decrease mortality rates or trim down D-to-B times, our study delves deeper, pinpointing specific components within chest pain centers leading to improved patient outcomes.Multiple studies have underscored the criticality of timely PCI treatment and rapid restoration of vessel patency, as they significantly reduce the mortality rate in STEMI patients (23–25).

### Additional challenges due to the COVID-19 pandemic

The ongoing pandemic posed an added challenge, necessitating a balance between urgent STEMI treatments and measures to contain the virus's spread (11). Our study suggests a strategic approach: swiftly identifying and prioritizing Killip ≥2 group STEMI patients and implementing additional precautions for the Killip 1 group.

### Further observations and recommendations

Our findings affirm that prolonged PCI informed consent and CL activation times can lead to increased mortality in Killip ≥2 patients, especially since these metrics correlate closely with D2B time (26). Enhancing public health awareness and fortifying physician-patient communication can potentially address these delays. Moreover, we observed no significant difference in mortality risks based on consultation methods (teleconsultation vs. on-site) or the varying professional backgrounds of medical personnel, highlighting the benefits of standardized procedures within chest pain centers (Supplementary Table 3).

### Limitations

The limitations of this study are mainly evident in the following aspects: This study is a retrospective investigation conducted within a specific time period and with a particular sample, which may introduce selection bias and restrict the generalizability and applicability of the findings; The data collection methods and metrics employed in the study may entail certain inaccuracies and subjectivity, potentially impacting the results.

### Future perspectives

Addressing the aforementioned limitations and shortcomings, future research should further explore the following areas: conduct prospective studies to minimize the possibility of omissions and biases; explore additional potential quality control indicators and factors to further enhance the effectiveness of emergency PCI procedures and patient outcomes; optimize the collaborative management of emergency PCI, including strengthening communication and coordination among physicians from diverse professional backgrounds and improving workflow across relevant departments to elevate the overall level of patient care.

## Conclusion

Our study identifies critical time-sensitive interventions, such as door-to-balloon, PCI informed consent, and time from diagnosis to DAPT loading dose, as significant determinants of mortality risk in STEMI patients, especially those with Killip ≥2 classification. Shortening specific treatment intervals markedly reduces this risk. Adherence to standardized documentation practices further mitigates mortality risks across all STEMI patients. Interestingly, consultation modalities and physicians' backgrounds showed no significant impact on outcomes. These findings highlight the necessity of tailored treatments based on Killip classification and the importance of standardized management in chest pain centers, especially during the COVID-19 pandemic.

## Data Availability

The datasets generated and analyzed during the current study are not publicly available because the database owner is reluctant to make them public but they are available from the corresponding author upon reasonable request. Requests to access these datasets should be directed to Lingling Zhang, linglingzhang0217@163.com.
