# Supplementary material for: Impact of chest pain center quality control indicators on mortality risk in ST-segment elevation myocardial infarction patients: a study based on Killip classification
Source: Front Cardiovasc Med. 2024 Jan 3;10:1243436. doi: 10.3389/fcvm.2023.1243436 (PMC10791892; doi:10.3389/fcvm.2023.1243436)
Supplement: Supplementary file 1 [file Table1.docx]

| **Supplementary Table 1. Baseline characteristics between quality control indicators and** death **grouped according to Killip** | | | | | | |
| --- | --- | --- | --- | --- | --- | --- |
|  | Killip class 1 Group | | | Killip class≥2 Group | | |
|  | no | yes | | no | yes | |
| N | 394 | 8 | | 235 | 27 | |
| **Quality control indexs as continuous variables** |  |  | |  |  | |
| FMC-to-ECG | 4.2 ± 3.5 | 5.0 ± 3.6 | | 4.5 ± 3.5 | 4.3 ± 3.5 | |
| FMC-to-loading dose DAPT | 31.9 ± 29.6 | 17.0 ± 10.4 | | 39.2 ± 35.0 | 44.7 ± 35.9 | |
| diagnosis-to-loading dose DAPT | 8.9 ± 8.4 | 4.8 ± 2.9 | | 9.9 ± 9.4 | 15.0 ± 11.9 | |
| diagnosis-to-the first intravenous heparin | 11.0 ± 4.5 | 9.4 ± 4.3 | | 11.5 ± 3.9 | 12.5 ± 4.9 | |
| Troponin report time | 17.6 ± 2.5 | 17.5 ± 1.9 | | 17.5 ± 2.2 | 17.9 ± 2.8 | |
| consultation time（notice to arrival） | 3.3 ± 1.7 | 4.4 ± 4.4 | | 3.2 ± 1.6 | 4.0 ± 2.3 | |
| D-to-B | 67.8 ± 25.3 | 69.1 ± 21.8 | | 70.2 ± 28.2 | 89.1 ± 33.1 | |
| total ischemic time (onset-to-reperfusion) | 380.6 ± 393.8 | 535.2 ± 465.8 | | 483.5 ± 692.3 | 403.2 ± 361.7 | |
| SO-to-FMC | 254.7 ± 365.7 | 411.1 ± 484.6 | | 323.4 ± 659.0 | 274.4 ± 344.4 | |
| FMC inhospital-to-start reperfusion | 22.3 ± 17.5 | 31.0 ± 29.1 | | 25.2 ± 20.4 | 28.3 ± 23.5 | |
| Diagnosis and treatment time in ED | 30.4 ± 23.5 | 32.8 ± 15.1 | | 28.9 ± 17.1 | 29.1 ± 15.9 | |
| FMC inhospital-to-notice consultation | 11.4 ± 15.2 | 14.9 ± 14.1 | | 10.8 ± 11.8 | 8.8 ± 6.0 | |
| Leave ED to arrive CL | 17.3 ± 23.5 | 15.2 ± 13.1 | | 21.6 ± 27.3 | 27.5 ± 33.6 | |
| PCI informed consent time | 11.1 ± 9.4 | 8.2 ± 5.5 | | 10.9 ± 11.0 | 20.0 ± 18.9 | |
| CL activation time | 13.6 ± 11.1 | 9.2 ± 7.4 | | 13.9 ± 10.0 | 18.1 ± 8.8 | |
| **Whether quality control indicators meet the standard** |  |  | |  |  | |
| FMC-to-ECG≥10min | 29 (7.4%) | 1 (12.5%) | | 24 (10.2%) | 2 (7.4%) | |
| FMC-to-ECG＜10min | 365 (92.6%) | 7 (87.5%) | | 211 (89.8%) | 25 (92.6%) | |
| FMC-to-loading dose DAPT≥30min | 145 (36.8%) | 2 (25.0%) | | 104 (44.3%) | 15 (55.6%) | |
| FMC-to-loading dose DAPT＜30min | 249 (63.2%) | 6 (75.0%) | | 131 (55.7%) | 12 (44.4%) | |
| diagnosis-to-loading dose DAPT≥10min | 144 (36.5%) | 1 (12.5%) | | 92 (39.1%) | 15 (55.6%) | |
| diagnosis-to-loading dose DAPT＜10min | 250 (63.5%) | 7 (87.5%) | | 143 (60.9%) | 12 (44.4%) | |
| diagnosis-to-the first intravenous heparin≥10min | 302 (76.6%) | 6 (75.0%) | | 207 (88.1%) | 24 (88.9%) | |
| diagnosis-to-the first intravenous heparin＜10min | 92 (23.4%) | | 2 (25.0%) | 28 (11.9%) | | 3 (11.1%) |
| Troponin report time＞20min | 9 (2.3%) | | 0 (0.0%) | 3 (1.3%) | | 2 (7.4%) |
| Troponin report time≤20min | 385 (97.7%) | | 8 (100.0%) | 232 (98.7%) | | 25 (92.6%) |
| consultation time（notice to arrival）≥10min | 4 (1.0%) | | 1 (12.5%) | 1 (0.4%) | | 1 (3.7%) |
| consultation time（notice to arrival）＜10min | 390 (99.0%) | | 7 (87.5%) | 234 (99.6%) | | 26 (96.3%) |
| D-to-B≥90min | 69 (17.5%) | | 1 (12.5%) | 50 (21.3%) | | 9 (33.3%) |
| D-to-B＜90min | 325 (82.5%) | | 7 (87.5%) | 185 (78.7%) | | 18 (66.7%) |
| total ischemic time (onset-to-reperfusion)≥120min | 350 (88.8%) | | 8 (100.0%) | 214 (91.1%) | | 24 (88.9%) |
| total ischemic time (onset-to-reperfusion)＜120min | 44 (11.2%) | | 0 (0.0%) | 21 (8.9%) | | 3 (11.1%) |
| SO-to-FMC≥90min | 246 (62.4%) | | 8 (100.0%) | 142 (60.4%) | | 18 (66.7%) |
| SO-to-FMC＜90min | 148 (37.6%) | | 0 (0.0%) | 93 (39.6%) | | 9 (33.3%) |
| FMC inhospital-to-start reperfusion≥30min | 73 (18.5%) | | 3 (37.5%) | 55 (23.4%) | | 8 (29.6%) |
| FMC inhospital-to-start reperfusion＜30min | 321 (81.5%) | | 5 (62.5%) | 180 (76.6%) | | 19 (70.4%) |
| Diagnosis and treatment time in ED≥30min | 154 (39.1%) | | 4 (50.0%) | 89 (37.9%) | | 12 (44.4%) |
| Diagnosis and treatment time in ED＜30min | 240 (60.9%) | | 4 (50.0%) | 146 (62.1%) | | 15 (55.6%) |
| FMC inhospital-to-notice consultation≥15min | 73 (18.5%) | | 3 (37.5%) | 33 (14.0%) | | 5 (18.5%) |
| FMC inhospital-to-notice consultation＜15min | 321 (81.5%) | | 5 (62.5%) | 202 (86.0%) | | 22 (81.5%) |
| Leave ED to arrive CL≥20min | 131 (33.2%) | | 3 (37.5%) | 89 (37.9%) | | 13 (48.1%) |
| Leave ED to arrive CL＜20min | 263 (66.8%) | | 5 (62.5%) | 146 (62.1%) | | 14 (51.9%) |
| PCI informed consent time≥20min | 74 (18.8%) | | 1 (12.5%) | 39 (16.6%) | | 11 (40.7%) |
| PCI informed consent time＜20min | 320 (81.2%) | | 7 (87.5%) | 196 (83.4%) | | 16 (59.3%) |
| CL activation time≥30min | 16 (4.1%) | | 0 (0.0%) | 15 (6.4%) | | 1 (3.7%) |
| CL activation time＜30min | 378 (95.9%) | | 8 (100.0%) | 220 (93.6%) | | 26 (96.3%) |
| Bold represent significant values (p < 0.05). ‡p < 0.001. §p < 0.05 | | | | | | |
| Abbreviations:CI:conﬁdence interval; MACE:major adverse cardiac events;CEP:composite endpoint; CCU:coronary care unit; FMC: first medical contact; ECG:electrocardiogram; DAPT:dual antiplatelet therapy; myocardial infarction; D-to-B:door-to-balloon ; SO:symptom onset; ED:emergency department; CL: catheter lab; PCI:percutaneous transluminal coronary intervention | | | | | | |
