# Supplementary material for: Impact of chest pain center quality control indicators on mortality risk in ST-segment elevation myocardial infarction patients: a study based on Killip classification
Source: Front Cardiovasc Med. 2024 Jan 3;10:1243436. doi: 10.3389/fcvm.2023.1243436 (PMC10791892; doi:10.3389/fcvm.2023.1243436)
Supplement: Supplementary file 2 [file Table2.docx]

| **Supplementary Table 2.Multivariate Cox proportional risk analysis of quality control indicators and death in core departments** | | | |
| --- | --- | --- | --- |
|  | N | N (Events/total Events%) | Multivariable adjusted Hazard ratio (95% CI) |
| **Pre-hospital emergency department** | | | |
| Fill in the chest pain form normatively | 129 | 6 (100.0%) | NA |
| Fill in the chest pain data platform normatively | 124 | 3 (50.0%) | 2.395 (0.419, 13.708) |
| FMC-ECG＜10min | 60 | 1 (33.3%) | NA |
| Pre-hospital ECG transmission | 113 | 4 (80.0%) | NA |
| Pre-hospital c00onsultation | 98 | 3 (75.0%) | NA |
| Bypass ED and CCU and D-to-B≤90min | 98 | 0 (0.0%) | NA |
| **Emergency department** | | | |
| Triage and establish archives normatively | 421 | 24 (100.0%) | NA |
| Fill in the chest pain form normatively | 418 | 15 (62.5%) | **0.312 (0.121, 0.799)§** |
| Fill in the chest pain data platform normatively | 412 | 18 (75.0%) | 0.748 (0.244, 2.288) |
| Write emergency medical records normatively | 421 | 18 (75.0%) | 1.009 (0.359, 2.833) |
| FMC-ECG＜10min | 419 | 22 (91.7%) | 0.309 (0.064, 1.497) |
| Troponin report time＜20min | 359 | 16 (72.7%) | 1.040 (0.381, 2.840) |
| diagnosis-to-loading dose DAPT＜10min | 271 | 8 (50.0%) | 0.349 (0.087, 1.399) |
| diagnosis-to-notice consultation＜5min | 347 | 20 (95.2%) | 0.299 (0.034, 2.593) |
| Bypass ED and D-to-B≤90min | 217 | 4 (36.4%) | 0.138 (0.018, 1.060) |
| **Catheter lab** | | | |
| Fill in the chest pain form normatively | 484 | 22 (95.7%) | 2.114 (0.249, 17.917) |
| Fill in the chest pain data platform normatively | 468 | 17 (73.9%) | 0.953 (0.334, 2.718) |
| Write operation record normatively | 484 | 23 (100.0%) | NA |
| CL activation time＜20min | 480 | 12 (52.2%) | 0.587 (0.227, 1.518) |
| Patients arrive at CL-to-guide wire passing＜30min | 483 | 6 (26.1%) | 0.491 (0.180, 1.341) |
| **Cardiovascular department** | | | |
| Fill in the chest pain form normatively | 501 | 16 (64.0%) | 1.518 (0.617, 3.736) |
| Fill in the chest pain data platform normatively | 484 | 14 (56.0%) | 0.770 (0.317, 1.869) |
| Write medical records normatively | 496 | 22 (88.0%) | 1.058 (0.280, 3.989) |
| Troponin report time＜20min | 28 | 0 (0.0%) | NA |
| diagnosis-to-loading dose DAPT＜10min | 46 | 2 (25.0%) | 0.838 (0.046, 15.307) |
| Write discharge medical record normatively | 495 | 10 (41.7%) | **0.071 (0.026, 0.190) ‡** |
| **Electrocardiology Department** | | | |
| ECG finish to diagnosis＜5min | 353 | 15 (78.9%) | 0.619 (0.175, 2.187) |
| **CPC consultants** | | | |
| Fill in the chest pain form normatively | 482 | 12 (52.2%) | 0.853 (0.336, 2.170) |
| consultation time（notice to arrival）＜10min | 472 | 21 (91.3%) | 0.169 (0.027, 1.073) |
| Bypass and D-to-B ≤90min | 475 | 7 (30.4%) | 0.839 (0.307, 2.292) |
| **‡p < 0.001. §p < 0.05** | | | |
| Multivariable adjust for: sex; age ; current smoker ; hyperlipidemia; hypertension; atrial fibrillation/atrial flutter; diabetes mellitus; stroke; renal insufficiency; admission systolic blood pressure; admission heart rate; maximum NT-proBNP; maximum troponin | | | |
| Abbreviations:N:number; CI:conﬁdence interval; MACE:major adverse cardiac events;CEP:composite endpoint; FMC: first medical contact; ECG:electrocardiogram; ED:emergency department; CCU:coronary care unit; D-to-B:door-to-balloon ;DAPT:dual antiplatelet therapy; CL: catheter lab; CPC:chest pain center; NA:not available | | | |
