# Supplementary material for: Impact of chest pain center quality control indicators on mortality risk in ST-segment elevation myocardial infarction patients: a study based on Killip classification
Source: Front Cardiovasc Med. 2024 Jan 3;10:1243436. doi: 10.3389/fcvm.2023.1243436 (PMC10791892; doi:10.3389/fcvm.2023.1243436)
Supplement: Supplementary file 3 [file Table3.docx]

| **Supplementary Table 3. Analysis of the differences between different consultation methods and different doctors in the key links in the CPC** | | | |
| --- | --- | --- | --- |
|  | N | Death | |
|  |  | N (Events/total Events%) | Multivariable adjusted Hazard ratio (95% CI) |
| Emergency department doctor | 664 |  |  |
| Cardiovascular medicine | 191 | 8 (22.9%) | Ref. |
| Emergency medicine | 346 | 21 (60.0%) | 1.578 (0.654, 3.803) |
| 0ther professional medicine | 127 | 6 (17.1%) | 1.057 (0.355, 3.147) |
| PCI surgeon | 664 |  |  |
| PCI surgeon is CCU doctor | 277 | 13 (37.1%) | Ref. |
| PCI surgeon is cardiology general ward doctor | 164 | 6 (17.1%) | 0.433 (0.150, 1.248) |
| PCI surgeon is CL doctor | 161 | 11 (31.4%) | 0.891 (0.383, 2.074) |
| Surgeon is Chief physician | 62 | 5 (14.3%) | 0.959 (0.296, 3.109) |
| CPC consultant classification | 664 |  |  |
| CPC consultant is cardiology general ward doctor | 324 | 13 (37.1%) | Ref. |
| CPC consultant is CCU doctor | 216 | 16 (45.7%) | 0.961 (0.432, 2.138) |
| CPC consultant is CL doctor | 124 | 6 (17.1%) | 0.602 (0.197, 1.842) |
| Consultation method | 664 |  |  |
| On-site consultation | 54 | 4(11.4%) | Ref. |
| Teleconsultation | 610 | 31(88.6%) | 1.027 (0.763, 1.383) |
| **‡p < 0.001. §p < 0.05** | | | |
| Multivariable adjust for: sex; age ; current smoker ; hyperlipidemia; hypertension; atrial fibrillation/atrial flutter; diabetes mellitus; stroke; renal insufficiency; admission systolic blood pressure; admission heart rate; maximum NT-proBNP; maximum troponin | | | |
| Abbreviations:CPC: chest pain Center; N:number; CI:conﬁdence interval; CCU:coronary care unit; CL: catheter lab; | | | |
